# The Great Imitator: A Case of Multisystem Sarcoidosis Involving the Genitourinary System

**DOI:** 10.7759/cureus.44401

**Published:** 2023-08-30

**Authors:** Zaki Alhashimalsayed, Karim Ladak, Sahar Al-Haddad, Christian Kraeker

**Affiliations:** 1 Department of Medicine, McMaster University, Hamilton, CAN; 2 Department of Pathology, McMaster University, Hamilton, CAN

**Keywords:** testicular, sarcoidosis, hypercalcemia, genitourinary, cancer

## Abstract

Sarcoidosis is a multisystem noncaseating granulomatous disease, which primarily involves the lungs, skin, and lymph nodes. In this case, we describe a 49-year-old Caucasian male presenting with weakness and symptomatic hypercalcemia. Initial workup revealed multiple testicular hypoechoic lesions on ultrasound and pulmonary nodules with hilar lymphadenopathy on a CT scan. Given the age of the patient, the initial differential diagnosis included lymphoma and testicular cancer. However, a lymph node biopsy confirmed the presence of noncaseating granulomas, and thus a diagnosis of multisystem sarcoidosis was made. Treatment with systemic steroids resulted in significant improvement, and he was initiated on methotrexate as a steroid-sparing agent. This case report details an unusual presentation of this multisystemic disease, which infrequently involves the genitourinary system, and presents a review of the literature on the “great imitator.”

## Introduction

Sarcoidosis is a multisystem noncaseating granulomatous disease that primarily involves the lungs, skin, and lymph nodes [1]. Urogenital sarcoidosis is very rare and is only seen in about 0.2% of cases. The epididymis is usually affected, followed by the testis and vas deferens [2]. Testicular cancer and sarcoidosis can present in a similar way, with the highest incidence occurring between the ages of 20 and 40 years old. Sarcoidosis is more common among African-American males, while testicular cancer predominantly affects Caucasian males in the U.S. population [3]. However, according to a Canadian national study with a primary focus on the epidemiology of sarcoidosis, it has been shown that sarcoidosis is more common among Canadian Caucasian males [4]. Testicular sarcoidosis has a variable presentation, ranging from unilateral painless mass to bilateral painful swelling, making the differential diagnosis more complex and sometimes leading to unnecessary treatment such as orchiectomy [1]. Here, we report a case involving a middle‑aged man presenting with a malignancy-like picture with constitutional symptoms who was found to have testicular sarcoidosis.

## Case presentation

**Figure 1:**
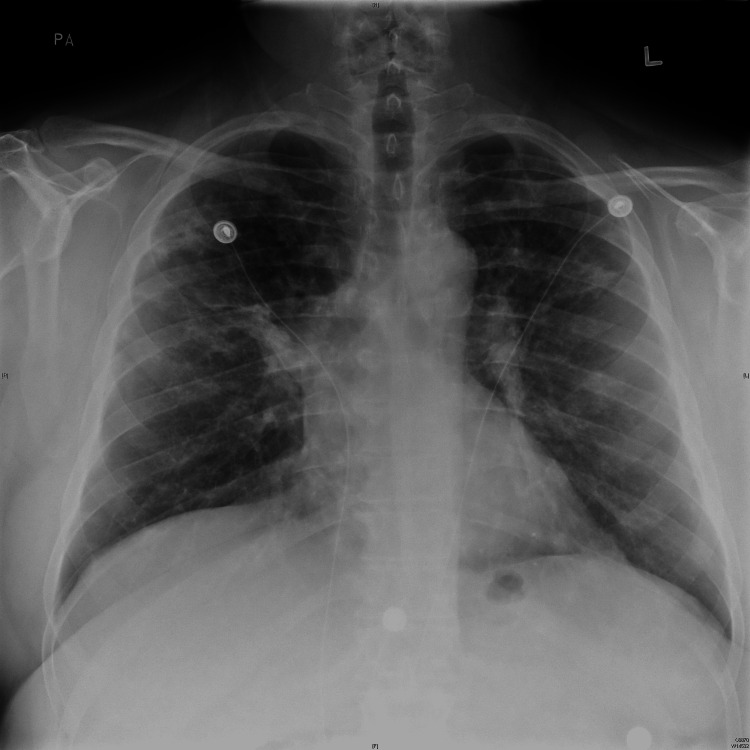
CXR showing numerous bilateral indistinct opacities suggestive of metastasis.

**Figure 2:**
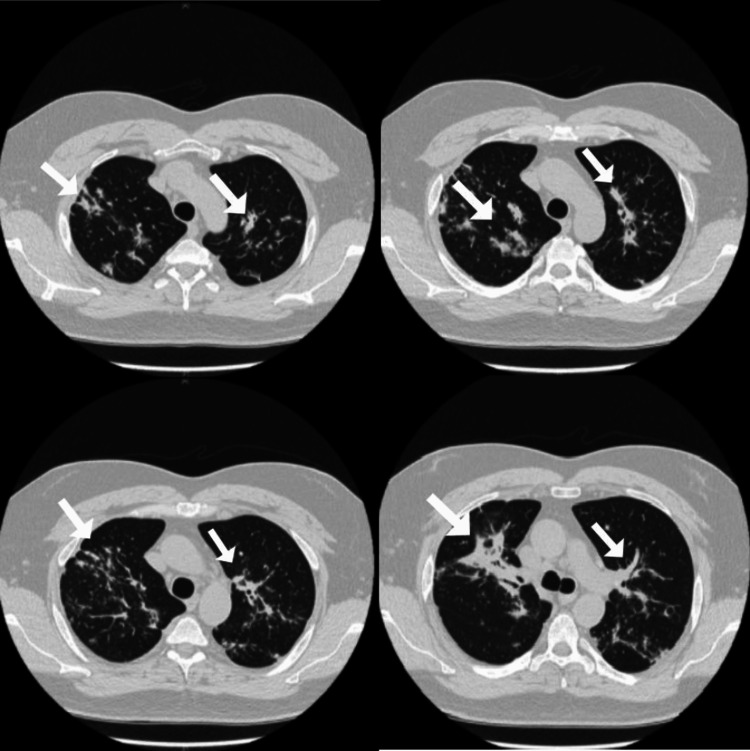
CT chest showing bilateral hilar lymph nodes with multiple irregular shaped nodules.

**Figure 3:**
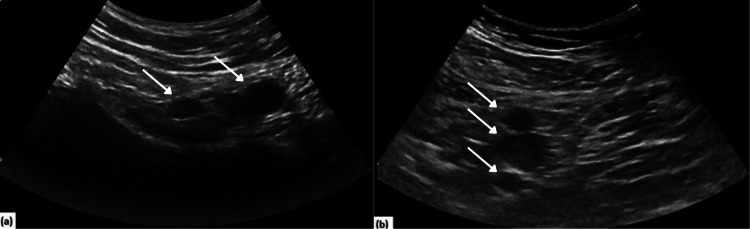
Ultrasound of the inguinal regions shows scattered hypoechoic macrolobulated lymph nodes. These lymph nodes have lost their expected fatty hilum. The largest lymph node on the right (a) measures 2.3 x 2.2 x 1.7 cm, and the one on the left (b) measures 2.4 x 1.8 x 1.1 cm.

A 49-year-old male, with a past medical history of gastroesophageal reflux disease (GERD) and vitiligo, presented with a three-month history of fatigue, generalized weakness, insomnia, and headache. He had pronounced anorexia and a 10-pound weight loss over the preceding four weeks. The patient had no family history of malignancy or inflammatory conditions. The physical examination was notable for a patchy, scaly, erythematous rash on the lateral right thigh and a palpable liver edge. Of note, the patient did not have testicular pain or swelling, and his testicular examination was unremarkable. Initial blood work showed acute kidney injury and hypercalcemia (Table 1). A chest X-ray was done on this patient, which showed multiple bilateral ill-defined opacities concerning metastasis (Figure 1). Based on his presentation and laboratory values, a whole-body computed tomography (CT) scan was completed and showed bilateral partially calcified enlarged hilar lymph nodes with multiple irregular-shaped nodules predominantly in the upper lobe and perilymphatic distribution (Figure 2). Additionally, there were multiple predominantly lytic skeletal lesions, specifically in the left sphenoid bone, and multiple enlarged abdominopelvic lymph nodes (Figure 3), which were concerning for metastases.

**Table 1:** Beta-hCG: beta-human chorionic gonadotropin, AFP: alpha-fetoprotein, PSA: Prostate-specific antigen.

Value (unit)	Result	Reference range
White blood cell count (x10 9/L)	8.0	4-11
Creatinine (umol/L)	225	60-110
Calcium (mmol/L)	2.95	2.10-2.60
Parathyroid hormone (pmol/L)	0.5	1.4-9.1
Phosphate (mmol/L)	0.96	0.90-1.52
Lactate dehydrogenase (U/L)	180	120-250
25-OH vitamin D (nmol/L)	58.4	125-175
Beta-hCG (IU/L)	<2	<5
AFP (ug/L)	3	≤6
PSA (ug/L)	0.52	<4.00

**Figure 4:**
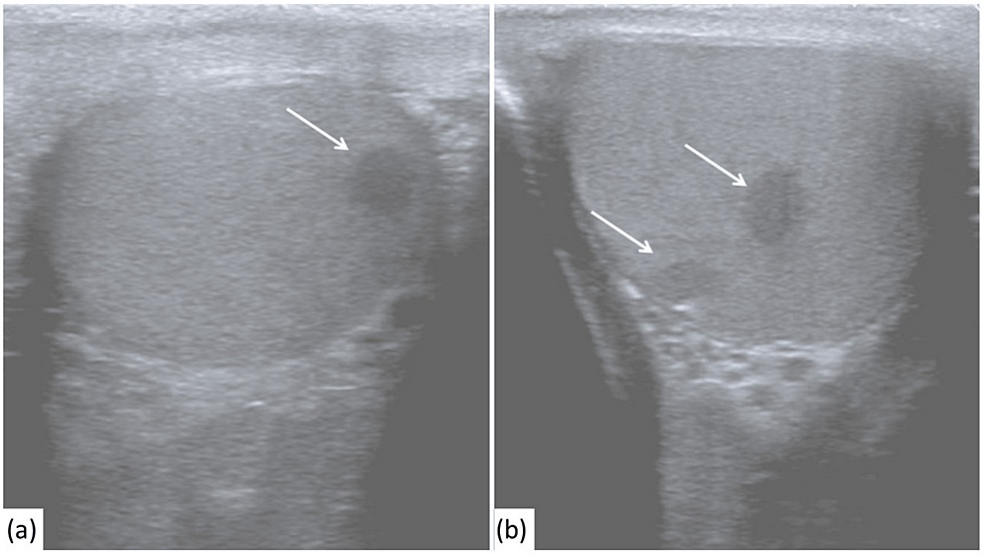
Scrotal ultrasonography with multiple rounded hypoechoic lesions in both testes (a, left testis; b, right testis).

**Figure 5:**
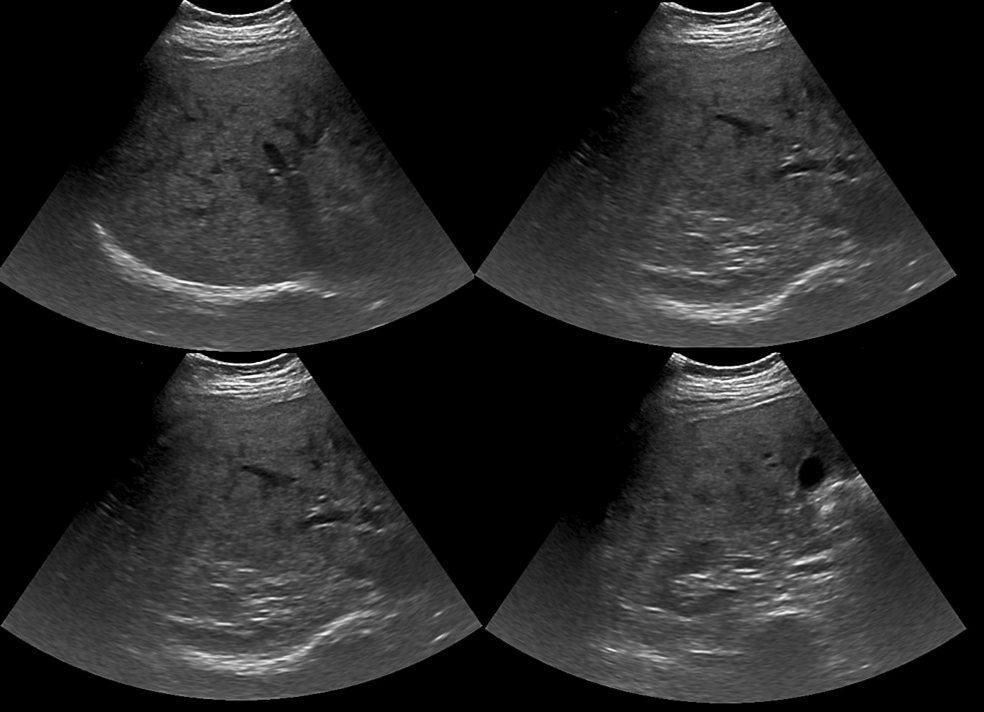
Ultrasound of the liver showing an enlarged liver measuring 17.0 cm with nodular and irregular contour. The parenchyma appears heterogeneous with diffusely increased echogenicity.

Due to high suspicion of testicular cancer, an ultrasound was completed and revealed normal-sized testes with multiple rounded hypoechoic lesions in both testes, much more on the right. The largest lesion measured 0.8 cm in the right upper pole and 0.7 cm in the right midpole. On the left, the largest lesion measured 0.6 cm in the mid-testis and 0.5 cm in the lower pole. The bilateral epididymides appeared normal with tiny epididymal cysts (Figure 4). Abdominal ultrasound showed nodular irregular hepatomegaly (Figure 5) with macrolobulated lymph nodes with lost fatty hila within the bilateral inguinal.

Based on the clinical presentation, initial laboratory workup, and imaging, along with a negative purified protein derivative (PPD) skin test, a preliminary differential diagnosis of lymphoma or testicular cancer was established. Tumor markers were sent for evaluation, all of which fell within the normal range (Table 1).

**Figure 6:**
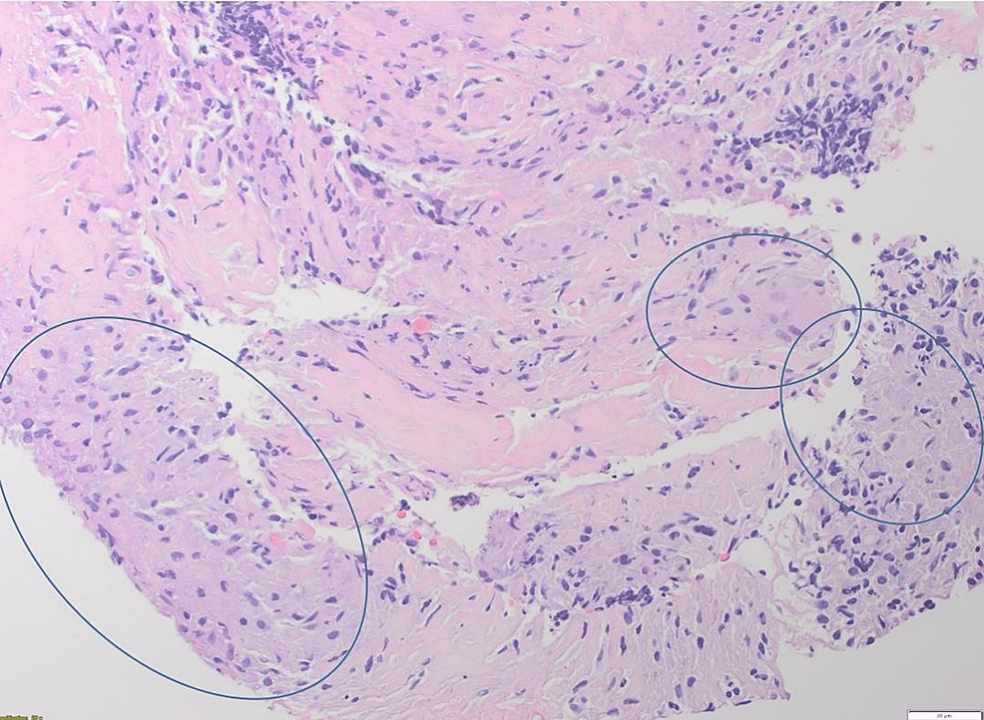
Multiple noncaseating granulomas (circles) in a background of dense fibrosis.

The patient was initially treated for his hypercalcemia and acute kidney injury with intravenous fluids and intravenous 90 mg of pamidronate. These interventions improved his clinical symptoms and renal function. Based on the absence of a clear diagnosis, biopsy of a right iliac lymph node was completed, and pathology showed multiple noncaseating granulomas in a background of dense fibrosis (Figure 6). Stains for acid-fast bacilli and fungi were negative. A diagnosis of multisystem sarcoidosis was made, and treatment with prednisone 40 mg PO daily was initiated with a gradual taper by 5 mg drop over every two weeks, until the patient is fully transitioned to a steroid-sparing agent only. This resulted in further symptomatic improvement back to the premorbid baseline. He continued to do well at his one-month follow-up after discharge from the hospital with normalization of his kidney function test and was started on methotrexate 25 mg PO weekly as a steroid-sparing agent in conjunction with folic acid. At the six-month follow-up, the patient had a cardiac MRI, which showed no cardiac involvement with normal ejection fraction and no myocardial scar or fibrosis. He is in clinical remission on methotrexate 15 mg PO weekly with good tolerance.

## Discussion

Sarcoidosis is a granulomatous disease that is commonly seen in young adults. It can affect any part of the human body, but most frequently involves the lungs and lymph nodes [5,6]. Genitourinary (GU) sarcoidosis is very rare and only affects 0.2% of all presenting cases [1]. The epididymis is the most involved GU organ, followed by the testicles [2,5]. Sarcoidosis exhibits a higher prevalence in African-American males within the U.S. demographic [3]. However, a cohort study using data from Ontario between 1996 and 2015 showed that the majority of patients with sarcoidosis in Canada are of Caucasian race [4]. Most patients present in an age group similar to that of testicular cancer. In addition, patients with testicular sarcoidosis are at an increased risk of getting testicular carcinoma. The two conditions can coexist, making the two difficult to distinguish. A study utilizing patient data from the Mayo Clinic found that individuals with confirmed testicular cancer have an incidence of sarcoidosis that is approximately 100 times higher [3]. However, more studies are needed to affirm this association. It has been suggested that inflammation caused by sarcoidosis can be potentially associated with neoplasm formation. There is also the possibility of impaired tumor antigen surveillance due to decreased cellular immunity in sarcoidosis [7]. It is very important to differentiate between testicular carcinoma and sarcoidosis. The prognosis, need for surgical intervention and chemotherapy, psychosocial impact, and future fertility are different between these two etiologies. Alongside sarcoidosis, it is important to consider and rule out other potential differential diagnoses such as fungal infection and tuberculosis. In exceedingly rare instances, sarcoidosis can also lead to the occurrence of caseating necrosis, a feature more commonly associated with infectious etiologies [8].

Imaging modalities, serum markers, and pathological samples of extra-testicular lesions can help differentiate cancer from sarcoidosis. For example, serum angiotensin-converting enzyme (ACE) is elevated in patients with active sarcoidosis in about 75% of all cases, but not in testicular cancer. Meanwhile, approximately 50% of patients with non-seminoma testicular cancers have elevated levels of serum AFP and beta-HCG (beta-hCG) [6]. An exception to that, however, would be AFP, which can be elevated in a patient with sarcoidosis and liver involvement. The case presented here was unique in that the patient did not have any specific systemic symptoms or testicular pain or swelling. Because of his age group and the findings of his chest imaging, a high suspicion of metastatic testicular cancer was present, and a scrotal ultrasound was done, which showed a testicular lesion, as described earlier, without epididymal involvement.

Sarcoidosis can be a self-remitting disease without any change in prognosis. Treatment using corticosteroids is warranted only in patients with symptoms impairing quality of life, persistent decline in pulmonary function, and cardiac, neurological, ocular, and disfiguring skin involvement, as well as other end organ failurea [9]. The use of corticosteroids has been associated with a good response in GU sarcoidosis. The effect of testicular sarcoidosis on fertility has not been examined, but fibrosis and occlusion of the epididymis can lead to azoospermia. Corticosteroids can improve sperm count by regressing the fibrotic obstructive process caused by the granuloma [1,10].

## Conclusions

A patient presenting with multisystemic and genitourinary sarcoidosis can easily mimic testicular cancer with metastasis to the lungs and lymph nodes. Such patients should be considered for sarcoidosis in the differential diagnosis as a potential etiology for their disease, especially if their tumor markers (AFP, beta-HCG, and LDH) are negative. Such consideration can lead to the avoidance of unnecessary orchiectomy if the correct diagnosis is made. Sarcoidosis can be a self-limiting disease without the need to subject patients to unnecessary treatment-related adverse effects. However, glucocorticoid is the treatment of choice when systemic therapy is indicated. In refractory cases or when steroid-sparing effects are desired, immunosuppressive and biological therapy, such as methotrexate and anti-TNF-alpha, can be added. Monitoring patients for potential treatment side effects and carefully striking a balance between the associated risks and benefits stands as a paramount consideration in the process of therapy selection.
